# From Office Environmental Stressors to Work Performance: The Role of Work Patterns

**DOI:** 10.3390/ijerph15081633

**Published:** 2018-08-02

**Authors:** Aida Soriano, Malgorzata W. Kozusznik, Jose M. Peiró

**Affiliations:** 1 Departamento de Psicología Social, IDOCAL, Universitat de València, Valencia 46010, Spain; aida.soriano@uv.es; 2 Research Group for Work, Organizational and Personnel Psychology (WOPP), Katholieke Universiteit Leuven, 3000 Leuven, Belgium; gosia.kozusznik@kuleuven.be; 3 Instituto Valenciano de Investigaciones Económicas (IVIE), Valencia 46020, Spain

**Keywords:** environmental stressors, health-related symptoms, negative emotions, performance, work patterns

## Abstract

*Background*: Different studies have shown a relationship between office environmental stressors and performance. However, studying environmental stress in the workplace requires analyzing more specific patterns to generate knowledge about the type of employees who are more or less vulnerable to environmental stressors. The present study analyzes the mediating role of health symptoms and negative emotions in the relationship between stressors and performance in different work patterns (task complexity and interactivity). *Methods*: There were 83 office workers (n = 603 time points) that took part in a diary study with multilevel design. *Results*: The appraisal of the environmental stressors is positively related to health-related symptoms, which in turn increase negative emotions, and then decrease the performance of workers who perform complex tasks and interact frequently with other people at work. This mediation is not significant when office workers do not interact frequently with other people at work and/or perform simple, rather than complex tasks. *Conclusions*: Work patterns play an important role when studying the mediating role of health-related symptoms and negative emotions in the relationship between the appraisal of environmental stressors and performance in office workers. In other words, employees in the ‘interactive and complex’ pattern are more vulnerable to the negative effects of office stressors on performance.

## 1. Introduction

More than 50% of the world’s workers spend long hours in offices [1], and this environment has an important impact on their health [2], well-being [3], and quality of life [4]. Indoor environmental quality may affect physiological processes such as thermal regulation [5] or immune system ailments, and disabilities that, in turn, can influence task performance, which might interact with other factors to affect overall productivity [6]. Therefore, it is important to consider the effects of indoor environmental quality on office workers’ health and performance [7], and to introduce improvements when necessary, because they might be beneficial for the employee and lead to financial gains [8] for the organization.

Work in offices can involve different activities that can be characterized by the amount of interaction with other people at work and the degree of complexity of the tasks. The combination of these two variables give rise to different work patterns. Researchers have shown that factors such as task complexity or interaction with other people may be potential boundary conditions for the effects of different work-related variables, influencing work outcomes [9]. However, the role of these work patterns in the relationship between the environmental stressors and health, well-being, and work performance has hardly been studied [10,11,12]. The purpose of the present study is to analyze the relationship between the appraisal of environmental stressors and performance, taking into account the mediating role of health-related symptoms and negative emotions, in different work patterns.

Given the importance of the workplace, it is surprising that most researchers have hardly considered the effects of the environmental stressors on productivity and well-being in different situations [5]. Understanding the mechanisms through which performance is affected would help us to better understand previous findings on the effects of the environmental stressors on performance [13]. In addition, knowing more about this effect in each work pattern would provide useful information regarding the most appropriate workplace design, to foster performance and ensure well-being at work in different types of office work.

### 1.1. The Impact of the Appraisal of Environmental Stressors on Workers’ Performance

Environmental psychology theory suggests that people’s environment has an impact on their behavior [14]. Environmental stressors are defined in terms of workers’ perceptions of discomfort in the indoor environment [15] (i.e., temperature and noise). There are many examples of situations where environmental stressors can influence human performance; however, some of the International Organization for Standardization (ISO) standards have considered human performance in a simple way. Therefore, a strategy for producing international standards was agreed on to consider human performance in physical environments [16]. The initial proposal for this framework considers three main reasons a physical environment might influence human performance [16], namely: (1) the physical environment’s interference with human function or activity; (2) the distraction caused by the physical environment and, hence, related to time off the task and work; and (3) the time involved in suspended work due to physical environments, beyond environmental health and safety limits. The interest in how the work environment affects employees has grown in recent decades in organizational psychology, with mounting evidence showing that the workspace affects the way people perform [17], and that the environmental stressors directly influence workers’ performance [18] and productivity [5] rates. For example, the results of several studies indicate that changes in temperature of a few degrees Celsius within the 18 °C to 30 °C range can significantly influence workers’ performance on several tasks, such as typewriting or reading speed and comprehension [18]. Along these lines, as discussed by the National Electrical Manufacturers Association [19], lighting has the theoretical potential to influence performance directly, because work performance depends on vision. Furthermore, in a New England survey described in the U.S. Environmental Protection Agency’s 1989 report to Congress, the average self-reported productivity loss due to poor indoor air quality was 3%. Finally, workers in open plan workspaces tend to cite noise as a cause of reduced productivity [20]. We consider, therefore, that offices’ environmental conditions will have an important impact on the work performance of their occupants. Taking into account the results obtained in previous research, we suggest that there should be a negative relationship between the appraisal of environmental stressors and office workers’ performance. Therefore, we formulate the following hypothesis:

**Hypothesis** **1.** The appraisal of environmental stressors will be negatively related to workers’ performance.

### 1.2. The Mediating Role of Negative Emotions between the Appraisal of Environmental Stressors and Performance

The environment can influence the emotions that people experience, and these emotions, in turn, can impact performance [21] (e.g., through approach–avoidance behavior [14]). In this line, being forced to work under unpleasant conditions can have negative consequences for affective well-being [22]. For example, temperatures that are too warm or too cool may produce negative emotions [23]. Moreover, office environment characteristics such as poor air quality or noise have been found to predict office workers’ negative emotions, such as anger, irritation, frustration, sadness or depression, worry, nervousness, and anxiety [24]. Additionally, the happy-productive worker thesis states that ‘happy’ workers perform better than ‘unhappy’ ones [25,26]. Therefore, employees with higher levels of negative emotions should perform worse than happy employees [27], because, when people feel worse than they usually do, they make less effort on their tasks [28] and achieve lower levels of task performance [29]. Moreover, people with low affective well-being tend to devise less imaginative solutions to problems [30]. With this in mind, we expect to find a relationship between the appraisal of environmental stressors and office workers’ negative emotions, and between office workers’ negative emotions and their performance. Therefore, we formulate the following hypothesis:

**Hypothesis** **2.** Office workers’ negative emotions will mediate the relationship between their appraisal of environmental stressors and their performance.

### 1.3. The Mediating Role of Health-Related Symptoms and Negative Emotions between the Appraisal of Environmental Stressors and Performance

Several building factors (e.g., ventilation system, rate of air ventilation, and indoor temperature) have been linked to the prevalence of acute building-related health symptoms experienced by the building’s occupants. These symptoms, which include irritation of the eyes, nose, and skin; headache; fatigue; and difficulty breathing, are most commonly reported by office workers [18]. In this regard, researchers increasingly find links between the employees’ health and aspects of the indoor environment at work, such as indoor air quality or lighting [31]. Improving the indoor work environment has been reported to result in a reduction in the number of physical complaints [5]. In turn, health-related symptoms, like headache or sore throat, are strongly related to people’s affectivity [32]. Some studies, although not referring directly to the health-related symptoms and negative emotions relationship, clearly suggest that there may be a positive relationship between these variables. They point out that self-report health measures show a pervasive mood of negative affectivity [33], and that health status is one of the most influential predictors of affective well-being, as people with an unfavorable self-reported health status have almost three times the odds of experiencing more negative emotions [34]. Taking these studies into account, and considering the happy-productive worker thesis [27], we expect that health-related symptoms will play a mediating role in the relationship between the appraisal of environmental stressors and negative emotions, which, in turn, will decrease performance. Therefore, we formulate the following hypothesis:

**Hypothesis** **3.** There will be an indirect effect of the appraisal of environmental stressors on performance through office workers’ health-related symptoms and subsequent negative emotions.

### 1.4. The Role of Work Patterns

Although different studies have shown a relationship between environmental stressors and performance [5,17,19,20], the relationship between some indoor conditions (i.e., lighting and temperature) and performance in typical office work still remains unclear [18]. Understanding stress in the workplace requires studying more specific patterns in order to generate knowledge about the type of employees that are more or less vulnerable to environmental stressors [35]. In the present study, we hypothesize that some psychosocial work characteristics may play an important role in this relationship. It has been established that work patterns (understood as configurations of work tasks, depending on their complexity and whether or not they imply interaction) can be relevant in different outcomes at work [9], establishing boundary conditions in these relationships. In other words, the work characteristics such as the complexity of tasks and interaction demands may moderate the relationship between environmental stressors and performance. Depending on the tasks employees are performing, they can be more or less affected by environmental factors [17]. Additionally, the detrimental effects of these demanding work patterns on well-being and performance may increase with longer working hours [36,37,38].

Firstly, the job stress theory [35] proposes that people’s appraisal of stressors depends on the balance of power between the environmental demands and the ability of the person to manage them. Secondly, activation theory [39] suggests that an individual’s activation level is directly related to the intensity, variation, uncertainty, and meaningfulness of the stimulus. This theory proposes that there is an optimal activation level, and when the activation is too low or too high, the workers’ performance decreases [40]. Therefore, we understand that, when an employee is working in a highly demanding environment because of environmental stressors and, simultaneously, has to carry out highly complex tasks that require additional mental effort, or has to interact with other people at work, the optimal activation level will be exceeded as the ability to manage those stressors may not be enough, which, in turn, will negatively affect the employee’s performance. With this in mind, each workspace can provide more or less support for people performing specific tasks that have specific environmental requirements. The more appropriate the space is for the task to be carried out, the more comfortable it is for the user, and the more it fosters task performance [17]. Dealing with a stressful workspace takes up the time and attention of its users, which, for employers, represents the time and attention taken from workers’ performance [17]. Moreover, the existence of a ‘cognitive reserve’ that allows people to maintain their performance during short exposure, even when indoor conditions are unfavorable [41], may not be enough to deal with complex tasks or to interact with other people at work at the same time.

Therefore, we suggest that work patterns should play an important moderating role in the relationship between the appraisal of environmental stressors and office workers’ performance through office workers’ health-related symptoms and negative emotions. Therefore, we formulate the following hypothesis:

**Hypothesis** **4.** The effects of the appraisal of environmental stressors on performance through office workers’ health-related symptoms and negative emotions will be stronger in the workers who perform complex tasks and interact frequently with other people at work, compared to those who perform simple tasks and/or do not interact frequently with other people at work.

Additionally, the relationship with the environment is constantly changing; environmental stressors may change across time and also patterns of coping with these stressors vary from one stressful encounter to another, and over time [42]. Furthermore, performance is also a dynamic phenomenon that may change across time and situation, as employees perform their tasks at work along the working day and week [43]. Thus, it is necessary to analyze the relationship between these variables using a methodological approach that considers states, which change across time. In this sense, a diary study allows us to focus on states [44] and to reduce measurement error (increasing validity and reliability) [45].

**Figure 1:**
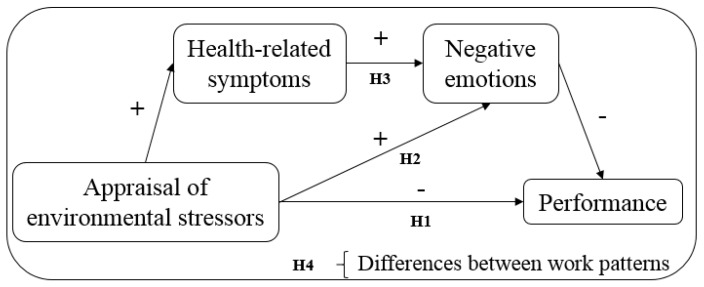
The proposed research model in this study.

Therefore, the main objective of this study is to analyze the relationship between the appraisal of environmental stressors and performance, taking into account the mediating role of health-related symptoms and negative emotions, in different work patterns. Figure 1 graphically represents the model to be tested in the study.

## 2. Materials and Methods

### 2.1. Sample and Procedure

In the present study, we collected data using a diary study and a baseline questionnaire completed by 83 office workers from five companies in the Valencian Community (Spain). Of the sample, 33% were men. The participants ranged in age from 20 to 62 years old (*M* = 39.67; *SD* = 8.84). Of the sample, 85% have at least a university degree.

The employees were asked to answer the questionnaire twice a day, once in the morning (after at least two hours at the workplace) and once in the afternoon, on four consecutive days. We aimed to collect the data from each of the employees in their offices at the same time in the morning and in the evening; however, because some of the respondents were away from the office during part of the workday, we failed to collect data at the 61 time points. Therefore, we obtained 603 data collection points. The employees’ work patterns were measured using baseline questionnaires administered between 1 and 4 days before the diary data collection week. The participants were informed that their participation in the study was voluntary, and they could withdraw from the study at any time. In addition, measures were taken to ensure the confidentiality of the data, and the study was approved by the institutional ethics committee.

### 2.2. Measures

The diary questionnaire assessed the state measures of appraisal of the environmental stressors, health-related symptoms, negative emotions, and performance. These measures reveal the participants’ levels on these characteristics on the specific occasions tested. Firstly, the ‘appraisal of environmental stressors’ was measured with a seven-item scale, based on a measure used by Andersson [46]. The person was asked to evaluate the extent to which he/she had been bothered by several factors at the workspace (noise, temperature, air quality, and light) in the past couple of hours (sample item, “temperature too high”). The response scale ranged from 1 (not at all) to 7 (very much). The mean Cronbach’s *a* for the scale at the eight time points was 0.70.

Secondly, ‘health-related symptoms’ (e.g., respiratory problems, headaches, and difficulties concentrating) due to the work environment were measured with a 10-item scale (sample item, “feeling heavy-headed”), which were adapted from Andersson [46]. The participants were asked to rate the extent to which they had experienced different health-related symptoms in the past couple of hours, on a response scale ranging from 1 (not at all) to 7 (very much). The mean Cronbach’s *a* for the scale at the eight time points was 0.84. 

Thirdly, ‘negative emotions’ were measured with a seven-item scale (sample item, “depressed”) [47,48]. The employees were asked to rate the extent to which they had experienced different negative emotions in the past couple of hours, using a response scale ranging from 1 (not at all) to 7 (very much). The mean Cronbach’s *a* for the scale at the eight time points was 0.84.

‘Work performance’ was measured with a six-item scale (sample item “now I fulfill all the requirements for my job”) assessing office workers’ in-role and extra-role performance [49,50]. The respondents were asked to evaluate to what extent they agreed with the different statements about their performance in the past couple of hours, using a response scale ranging from 1 (not at all) to 7 (very much). The mean Cronbach’s *a* for the scale at the eight time points was 0.76.

Finally, ‘work patterns’ were measured by two items (sample item, “how often your job require do complex tasks?”), referring to the frequency of performing complex tasks and the frequency of interacting with other people at work. The response scale ranged from 1 (never) to 4 (very often). 

### 2.3. Data Analysis

In the first part of the analyses, the sample was divided into groups using two-step cluster analysis in SPSS v.22 (SPSS Inc., Chicago, IL, USA), and considering two variables (i.e., work patterns), namely the task complexity and interaction with other people at work. This method is derived from a probabilistic model in which the distance between two clusters is equivalent to the decrease in the log-likelihood function as a result of merging [51]. Its algorithm is based on a two-step approach; firstly, it uses a similar procedure to the k-means algorithm; secondly, considering these results, a modified hierarchical agglomerative clustering procedure is carried out that combines the objects sequentially to form homogenous clusters. This method offers fit information such as the Bayesian Information Criterion (BIC), as well as information about the importance of each variable in the construction of a specific cluster [52], which is an additional attractive feature of the two-step cluster method compared to traditional clustering methods. As all of the variables used in this study were independent and had a normal distribution (kurtosis and skewness ±2, [53]), we used the log-likelihood approach [54].

In the second part of the analyses, we carried out multi-level structural equation modeling (MSEM) to determine the relationships between the variables of interest in the different work patterns. To this end, we used a diary and multi-level design, as for each employee, the data on two levels were available, namely the time-level and the person-level, with the time-level data being nested within the person-level data. As the following section shows, because only seven subjects belonged to the first work pattern, we continued with the MSEM analysis, taking into account only the participants who formed part of the other three work patterns. This led to a two-level model with the repeated measures at the first level (N = 549 study occasions) and the individual participants at the second level (N = 76 participants). As we were interested in the relationships between variables at the individual level, we focused on assessing the relationships at the ‘person level’ (i.e., level-2 or between-level), which takes into account between-person variations. A diary study allows us to focus on states, which change across time and reflect how an individual feels at certain points in time [44]. In order to carry out multi-level, multi-group structural equation modeling, we used MPlus software [55]. To test the significance of the indirect effects, we produced confidence intervals using the Monte Carlo method for assessing mediation (MCMAM) [56] with 20,000 repetitions.

In order to assess the model fit, we examined the root mean square error of approximation (RMSEA), comparative fit index (CFI), Tucker–Lewis index (TLI), and root mean square residual (SRMR) goodness of fit statistics. For the ML method, a cutoff value close to 0.06 for RMSEA, a cutoff value close to 0.95 for CFI and TLI, and a cutoff value close to 0.08 for SRMR are necessary before we can conclude that there is a relatively good fit between the hypothesized model and the observed data [57].

## 3. Results

### 3.1. Two-step Cluster Analyses

The auto-clustering algorithm of the two-step cluster analyses indicated that a four-cluster solution was the best model because it minimized the BIC value (101.860) and the change in them between adjacent numbers of clusters selection criteria (−3.222). All of the predictors (task complexity and interaction) explained at least 78% of the cluster analysis results, and the average silhouette was 0.6. Four clusters emerged as follows: (1) employees who work alone and perform simple tasks (i.e., ‘alone, low complexity’); (2) employees who sometimes interact with other people at work and perform complex tasks (i.e., ‘middle interactive, high complexity’); (3) employees who frequently interact with others and perform simple tasks (i.e., ‘high interactive, low complexity’); and (4) employees who frequently interact with other people at work and perform complex tasks (i.e., ‘high interactive, high complexity’). The first group (‘alone, low complexity’) was not taken into account in the following analyses as a result of the low number of participants (n = 7). Therefore, the final sample was composed of 76 workers divided into three groups (work patterns). The descriptive analyses are shown in Table 1. We carried out variance analyses (ANOVA) and chi^2^ significance tests for the differences in the demographic variables between the clusters in each combination. No differences were found between groups, except for the sex variable, as the ‘middle interactive, high complexity’ pattern has more men than the ‘high interactive, high complexity pattern’ (*p* = 0.03).

### 3.2. Multi-Level, Multi-Group Structural, Equation Modeling

Table 2 presents the descriptive statistics for the levels of the variables of interest in the current study. To test the predictive validity of the coping factors at both levels of the nested data structure, structural equation modeling for multi-level data (MSEM) was used to predict office workers’ performance. The model fit was excellent, as follows: RMSEA = 0.000, CFI = 1.000, TLI = 1.075, SRMR (Within/Between) = 0.020/0.032.

**Figure 2:**
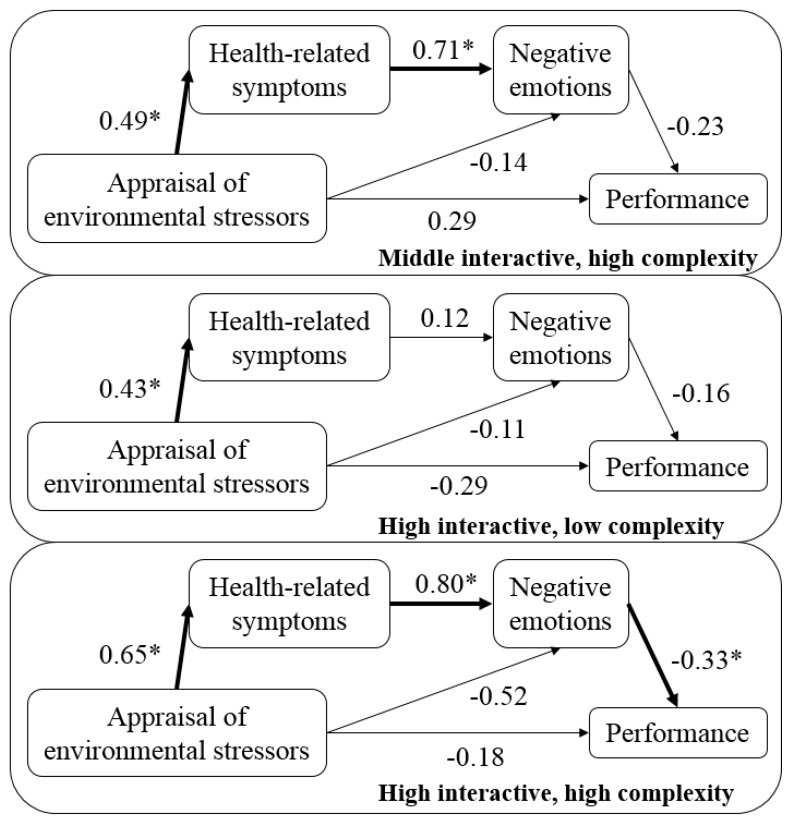
Multi-level multi-group structural equation modeling. * *p* < 0.05.

Figure 2 presents the results of the Multi-level multi-group structural equation modeling analyses, and Table 3 presents the results of the Monte Carlo method for assessing the mediation for the different groups.

Regarding the direct effect of the appraisal of environmental stressors on workers’ performance, the results do not provide support for Hypothesis 1, as they were not significant for any of the work patterns (*p* > 0.05). The same results were obtained for the mediating role of negative emotions, as the results do not support hypothesis 2 for the ‘middle interactive, high complexity’ group [LL −0.03; UL 0.23], the ‘high interactive, low complexity’ group [LL −0.09; UL 0.15], or the ‘high interactive, high complexity’ group [LL −0.01; UL 0.59]. In the case of hypothesis 3, the results give partial support, as a significant indirect effect through health-related symptoms and negative emotions was found for the ‘high interactive, high complexity’ pattern [LL −0.51; UL −0.01], but not for the ‘middle interactive, high complexity’ pattern [LL −0.24; UL 0.03] or the ‘high interactive, low complexity’ pattern [LL −0.10; UL 0.04]. Therefore, the results support hypothesis 4, as the effects of the appraisal of environmental stressors on performance through office workers’ health-related symptoms and negative emotions were stronger in workers who performed complex tasks and frequently interacted with other people at work, compared to those who performed simple tasks and/or did not have to interact frequently with other people at work.

## 4. Discussion

The aim of this study was to analyze the relationship between the appraisal of environmental stressors and performance, taking into account the mediating role of health-related symptoms and negative emotions, in different work patterns. Regarding the direct effect of the appraisal of environmental stressors on workers’ performance, the results show that there is no significant direct effect in any of the work patterns. The same results were obtained for the mediating role of negative emotions. For the double mediation, the results showed a significant indirect effect through health-related symptoms and negative emotions for the ‘high interactive, high complexity’ pattern, but not for the ‘middle interactive, high complexity’ pattern, or the ‘high interactive, low complexity’ pattern. Therefore, the effects of the appraisal of environmental stressors on performance through office workers’ health-related symptoms and negative emotions were stronger for the ‘high interactive, high complexity’ work pattern than for the other patterns.

Thus, in contrast to the literature [5,18], the results show that the direct relationship between the appraisal of environmental stressors and performance was not significant. With regard to the indirect effect, on the one hand, and in contrast to previous studies [23,24,27], the indirect relationship between the appraisal of environmental stressors and performance through negative emotions was not significant. On the other hand, as expected, taking into account the existing literature [5,27,29,34], the double mediation through health-related symptoms and negative emotions, in that order, was significant for the ‘high interactive, high complexity’ pattern, but not for the ‘middle interactive, high complexity’ or ‘high interactive, low complexity’ patterns. These results can be explained based on the activation theory [39] and the job stress theory [35]. The first one says that there is an optimal activation level, and when this level is exceeded (e.g., appraisal of environmental stressors, task complexity, and interaction with other people at work), workers’ performance declines [13]. The second one, proposes that the workers’ appraisal of stress depends on the balance of power between the environmental demands and the ability of the person to manage them, thus to many demands (e.g., appraisal of environmental stressors, task complexity, and interaction with other people at work) may decrease performance.

The main contribution of this study is that it analyzes the role of work patterns in the relationships between the appraisal of environmental stressors, office workers’ health-related symptoms, negative emotions, and performance. Furthermore, the present study uses a diary study design that allows us to pay attention to states, which vary over time and reflect how an individual feels at certain points in time, rather than understanding well-being as an overall judgement related to long periods, disregarding its variability. Although this study did not describe changes across time, it allows us to control the variability of these variables by reducing the measurement error (increasing validity and reliability) [44].

Despite its contributions, the present study has some limitations. Firstly, in the present study, we used self-reported measures of state performance. Even though strong congruence has been found between company records and workers’ self-reports [58], and workers’ self-reports are more controversial in the case of general judgments than on diary-state measures [59], future studies could compare these self-reports to other more objective measures. Secondly, as a result of the sample limitations, we have not taken the work pattern “Alone, low complexity” into account, and so, it would be necessary to increase the sample and investigate these relationships in the four work patterns in future studies. The results of the present study suggest that future research should investigate these relationships in different occupational samples, and consider other relevant aspects that may play an important role in work pattern configurations. Thirdly, future studies should consider the effect of the moment when the data collection took place (i.e., season), because the workplace temperature may vary depending on this moment. However, the temperature in Valencia is quite similar during the months when we collected the data [60]. Finally, we have to recognize that associations between environmental stressors and performance are extremely complex. For example, the present study does not take into account how many hours the employees spend at their workplace under the influence of this environment. Different studies have shown the effect of long work hours on workers’ performance and well-being [36,37,38], probably due to the impact on fatigue [61].

Our findings have important theoretical implications. Firstly, in this study, we take into account different constructs that play a mediating role in the relationship between the appraisal of environmental stressors and performance. Secondly, we consider the role of work patterns in these associations, which, until now, had hardly been examined. This concept has been fruitful and useful, with both theoretical and practical significance. Furthermore, we understand that the appraisal of environmental stressors, health-related symptoms, negative emotions, and performance vary over time, and our study design allows us to study these changes across time.

This study also has important practical implications. Its results highlight the importance of providing an adequate workspace, in terms of the indoor environment, in different office worker situations. Moreover, they offer important information about the implications of exposure to environmental stressors for office workers’ performance and well-being. Furthermore, this study can provide important information for supervisors and managers regarding human resources practices for different groups of employees. Taking work patterns into account can be useful in time management or task assignment, considering the specific aspects of each task (task complexity and task interaction). Thus, this study emphasizes the importance of optimizing environmental stressors, highlighting that workers who perform complex tasks and interact with other people at work are more impaired by environmental stressors. Therefore, future studies should analyze how organizations or supervisors might support employees in general, and this group in particular, to enhance their well-being and performance. Optimizing environmental stressors is an important first step; however, it may also be necessary to take other factors into account to compensate for these highly demanding tasks (i.e., offering adequate workspaces, such as individual offices that facilitate the performance of these tasks). The study also provides orientations for organizational psychologists; they can organize activities such as training courses that consider the specific characteristics of each group of office workers. As a result of the long hours office workers spend in their workplaces, and the impact these environments have on their health [2], it is necessary to recognize the relevance and specific characteristics of the employees’ health and well-being in different types of office work, in order to understand how we should approach this topic and improve the workers’ health while ensuring their performance.

## 5. Conclusions

This study highlights the important role of work patterns when studying the mediating effect of health-related symptoms and negative emotions in the relationship between the appraisal of environmental stressors and performance in office workers. This approach to the study of the relationships between different work-related variables and their boundary conditions according to different work patterns is novel and reveals their distinct characteristics and implications. Knowledge about their different characteristics and implications is important in order to carry out preventive actions that can foster performance and well-being at work in office workers’ different situations.

## Figures and Tables

**Table 1:** Work patterns: sample characteristics.

	Middle Interactive, High Complexity (n = 32) n (%)	High Interactive, Low Complexity (n = 23) n (%)	High Interactive, High Complexity (n = 21) n (%)	Chi^2^	*p*
Age ^1^	41.09 (8.14)	37.96 (9.86)	38.67 (8.05)	1.001	0.37
Sex ^2^				6.693	0.03
Female	17 (22.4%)	17 (22.4%)	18 (23.7%)		
Male	15 (19.7%)	6 (7.9%)	3 (3.9%)		
Highest educational level reached ^2^					
High school	1 (1.3%)	0 (0.0%)	0 (0.0%)	5.737	0.67
Professional training	4 (5.3%)	3 (3.9%)	3 (3.9%)		
University degree (Graduated)	8 (10.5%)	7 (9.2%)	4 (5.3%)		
University degree (MA, Msc)	18 (23.7%)	11 (14.5%)	10 (13.2%)		
PhD	1 (1.3%)	2 (2.6%)	4 (5.3%)		
Job level ^2^				12.923	0.11
Manager	1 (1.3%)	0 (0.0%)	2 (2.6%)		
Highly-qualified professional	12 (15.8%)	5 (6.6%)	10 (13.2%)		
Technician	8 (10.5%)	7 (9.2%)	5 (6.6%)		
Administrative	10 (13.2%)	11 (14.5%)	2 (2.6%)		
Junior employees	0 (0.0%)	0 (0.0%)	0 (0.0%)		
Other	1 (1.3%)	0 (0.0%)	2 (2.6%)		
Marital status ^2^				2.945	0.56
Single	10 (13.2%)	4 (5.3%)	6 (7.9%)		
Married/living with partner	21 (27.6%)	19 (25.0%)	15 (19.7%)		
Widowed	0 (0.0%)	0 (0.0%)	0 (0.0%)		
Separated/divorced	1 (1.3%)	0 (0.0%)	0 (0.0%)		
Salary ^2^				12.397	0.25
Less than 600€	2 (2.6%)	1 (1.3%)	2 (2.6%)		
600€–1000€	1 (1.3%)	2 (2.6%)	1 (1.3%)		
1000€–1499€	14 (18.4%)	11 (14.5%)	7 (9.2%)		
1500€–1999€	5 (6.6%)	8 (10.5%)	7 (9.2%)		
2000€–3000€	10 (13.2%)	1 (1.3%)	3 (3.9%)		
More than 3000€	0 (0.0%)	0 (0.0%)	1 (1.3%)		

Note. *n* = 76; ^1^ Means, standard deviations, and variance analyses (ANOVA). ^2^ The number in parentheses represents the percentage of the total.

**Table 2:** Descriptive statistics for the levels of the variables of interest in the current study.

	Middle Interactive, High Complexity		High Interactive, Low Complexity		High Interactive, High Complexity	
	*M*	*SD*	*M*	*SD*	*M*	*SD*
Appraisal of environmental stressors t1	2.13	0.96	1.92	0.74	2.31	0.83
Appraisal of environmental stressors t2	2.35	0.99	2.19	0.90	2.30	0.90
Appraisal of environmental stressors t3	1.96	0.95	1.88	0.75	2.17	0.85
Appraisal of environmental stressors t4	2.12	1.09	1.95	0.86	2.27	1.02
Appraisal of environmental stressors t5	1.94	0.96	1.99	1.02	2.00	0.92
Appraisal of environmental stressors t6	2.14	1.06	2.12	1.00	2.16	0.97
Appraisal of environmental stressors t7	1.94	1.00	1.98	0.95	2.02	0.76
Appraisal of environmental stressors t8	2.11	1.14	2.06	1.00	2.09	0.85
Health-related symptoms t1	1.83	0.94	1.87	0.81	2.08	0.91
Health-related symptoms t2	2.01	0.99	2.18	0.95	2.25	1.02
Health-related symptoms t3	1.54	0.65	1.57	0.66	1.83	0.88
Health-related symptoms t4	1.80	0.85	1.87	0.85	2.35	1.32
Health-related symptoms t5	1.55	0.64	1.70	0.57	1.98	1.02
Health-related symptoms t6	1.80	0.91	2.03	0.97	2.25	1.02
Health-related symptoms t7	1.56	0.70	1.53	0.62	1.89	1.11
Health-related symptoms t8	1.80	0.87	1.80	0.84	2.09	0.98
Negative emotions t1	2.14	1.19	2.32	1.16	2.40	1.13
Negative emotions t2	2.15	1.14	2.24	1.10	2.25	0.91
Negative emotions t3	1.85	0.81	1.97	1.01	2.14	1.36
Negative emotions t4	2.02	1.00	2.13	1.36	2.34	1.20
Negative emotions t5	1.90	0.97	1.76	0.73	1.99	1.17
Negative emotions t6	2.22	1.13	1.86	0.58	1.95	0.77
Negative emotions t7	1.84	0.93	1.64	0.72	1.77	0.88
Negative emotions t8	2.06	1.20	1.87	1.03	2.01	0.94
Performance t1	5.08	0.96	4.88	0.79	5.23	1.16
Performance t2	5.06	0.99	4.76	1.04	5.09	0.96
Performance t3	5.06	0.96	4.77	1.25	4.92	1.07
Performance t4	5.06	1.10	5.00	1.09	5.33	1.01
Performance t5	5.02	1.07	4.99	0.90	5.21	0.99
Performance t6	5.07	1.09	4.81	1.04	5.30	1.08
Performance t7	5.29	0.97	4.96	1.08	4.82	0.90
Performance t8	5.10	1.07	4.84	1.11	4.82	0.88

**Table 3:** Monte Carlo simulation for the indirect effects.

	Middle Interactive, High Complexity		High Interactive, Low Complexity		High Interactive, High Complexity	
	Lower	Upper	Lower	Upper	Lower	Upper
Negative emotions path	−0.03	0.23	−0.09	0.15	−0.01	0.59
Health-related symptoms—negative emotions path	−0.24	0.03	−0.10	0.04	−0.51	−0.01
